# Metals in Cyanobacteria: Analysis of the Copper, Nickel, Cobalt and Arsenic Homeostasis Mechanisms

**DOI:** 10.3390/life4040865

**Published:** 2014-12-09

**Authors:** María José Huertas, Luis López-Maury, Joaquín Giner-Lamia, Ana María Sánchez-Riego, Francisco Javier Florencio

**Affiliations:** 1 Instituto de Bioquímica Vegetal y Fotosíntesis, Universidad de Sevilla-CSIC, Américo Vespucio 49, E-41092 Sevilla, Spain; E-Mails: mjhuertas@us.es (M.J.H.); llopez1@us.es (L.L.-M.); amriego@ibvf.csic.es (A.M.S.-R.); 2 Systems Biology and Bioinformatics Laboratory, IBB-CBME, University of Algarve, Campus de Gambelas, 8005-139 Faro, Portugal; E-Mail: ginerorama@gmail.com

**Keywords:** metal homeostasis, copper, nickel, cobalt, arsenic, metal transport

## Abstract

Traces of metal are required for fundamental biochemical processes, such as photosynthesis and respiration. Cyanobacteria metal homeostasis acquires an important role because the photosynthetic machinery imposes a high demand for metals, making them a limiting factor for cyanobacteria, especially in the open oceans. On the other hand, in the last two centuries, the metal concentrations in marine environments and lake sediments have increased as a result of several industrial activities. In all cases, cells have to tightly regulate uptake to maintain their intracellular concentrations below toxic levels. Mechanisms to obtain metal under limiting conditions and to protect cells from an excess of metals are present in cyanobacteria. Understanding metal homeostasis in cyanobacteria and the proteins involved will help to evaluate the use of these microorganisms in metal bioremediation. Furthermore, it will also help to understand how metal availability impacts primary production in the oceans. In this review, we will focus on copper, nickel, cobalt and arsenic (a toxic metalloid) metabolism, which has been mainly analyzed in model cyanobacterium *Synechocystis* sp. PCC 6803.

## 1. Introduction

Metals are basic micronutrients for all living organisms and play essential roles in fundamental biochemical processes that sustain life on Earth, like respiration and photosynthesis. They are critical for correct folding and functioning of many proteins, but many metal-containing proteins remain unknown, at least in their function [1]. Recently, it has been shown that up to a third of the total proteome of microbes contains a metal cofactor [2], but there are limitations in metallo-protein prediction that make it difficult to predict if a protein will contain metals or not. *In vitro*, the majority of metalloproteins will bind metals with an order of affinity following the Irving-Williams series for divalent cations (Mg^2+^ < Ca^2+^ < Mn^2+^ < Fe^2+^ < Co^2+^ < Ni^2+^ < Cu^2+^ > Zn^2+^), and therefore, cells have a great challenge delivering the right metal to the right proteins [1]. Furthermore, when the concentrations of metals increase in the cell, they undergo undesirable redox reactions or bind inappropriately to the metal binding sites of several enzymes, leading to toxic effects [3,4]. Thus, the acquisition of metals has to be strictly balanced in the cells, and hence, all organisms have evolved to respond to the bioavailability of metals in order to sustain metal homeostasis.

The occurrence of heavy metals in the biosphere is a natural phenomenon, as they are elements that cannot be destroyed or degraded. In the last century, heavy metals have accumulated in the environment as a result of their enhanced utilization in several industrial activities, including their extraction in mining processes. Their management is a major global concern, because of their toxicity, and a threat to both human life and biosphere conservation. Heavy metal accumulation has been especially intense in marine environments [5] and lake sediments [6]. In metal-polluted environments, metal homeostasis is important to protect cells from metal overload, which will cause oxidative stress, lipid peroxidation, damage of nucleic acids and the inhibition of essential processes, like respiration, carbon metabolism and photosynthesis [7,8]. One of the approaches to control the presence of heavy metals involves the use of microorganisms to remediate these contaminants [8,9].

Cyanobacteria are the only group of prokaryotes capable of performing oxygenic photosynthesis and spread out in almost any ecological niche from fresh and salt water to terrestrial and extreme environments, including metal-contaminated areas [10]. Metal homeostasis is especially important in cyanobacteria, because the photosynthetic machinery imposes a high demand for metals, acting as cofactors of several proteins [11]. Metals play essential roles in cyanobacterial metabolism, for example: iron is required as a cofactor for all three photosynthetic electron transfer chain complexes [7]; Mn is specifically required for PSII (Photosystem II) function [12]; Mg is coordinated at the center of the chlorophyll ring [13]; and zinc is required for carbonic anhydrase [14]. In fact, the distribution and abundance of marine cyanobacteria and, therefore, phytoplankton’s productivity in natural habitats is often determined by the metal bioavailability, especially by iron, but also by copper, cobalt, zinc or nickel [15,16,17]. This availability can limit cyanobacterial growth, not only because of scarcity, but also due to toxic effects, as it has been shown that different clades and species have different metal susceptibilities [15,16].

Adaptation to high metal concentration in cyanobacteria includes mechanisms, such as their ability to excrete to the media heavy metal ligands, like siderophores [18,19] or extracellular polymeric substances (EPS) [9,20,21,22], the production of metallothioneins [23,24,25,26,27] or induction of metal transporters [26,28,29,30,31,32,33,34]. Understanding the mechanisms of how metals interact with cyanobacteria in natural systems will allow the use of these organisms as promising detector systems for metal bioremediation. Furthermore, knowing the influence of metal availability in the cyanobacterial distribution in the oceans, where they have an important role in global primary production, therefore, could have a great impact on our understanding of the global carbon cycle. It will also help to identify in which conditions cyanobacteria growth is favored, allowing the design of optimized processes to use them as biofactories [35]. In this review, we will focus on the more recent advances in cyanobacterial homeostasis of the metals, copper, nickel and cobalt, and the metalloid, arsenic. Other metals, like iron, zinc or manganese, have been recently reviewed and will not be included in this study [7,15,36].

## 2. Copper, an Essential Element for Photosynthesis and Respiration

Copper is an essential micro-nutrient that is required as a cofactor for a number of cuproenzymes, including amine oxidases, cytochrome c oxidases, laccases, methane monooxygenases, multicopper oxidases, nitrite oxidases, plastocyanin, superoxide dismutases and tyrosinases. These proteins are involved in diverse cellular processes, such as energy transduction, iron mobilization and oxidative stress response [37,38,39]. The ability of copper to alternate between its cuprous Cu(I) and cupric Cu(II) oxidation states makes it an ideal biological cofactor, especially for processes in which the electron transfer is a key factor, such as photosynthesis and respiration. However, the two oxidation states of copper not only allow its participation in essential redox reactions, but also allow it to catalyze the production of reactive oxygen species (ROS) through the Fenton and Haber–Weiss reactions, which leads to severe damage to lipids, proteins, DNA and other cytoplasmic molecules [38]. Furthermore, an excess of copper competes with other metals for their binding sites in proteins following the Irving-Williams series [1], resulting in a perturbation of protein function and, in some cases, protein degradation. Recently, it has been proposed that the main target for copper toxicity in *Escherichia coli*, *Bacillus subtilis* and *Synechocystis* sp. PCC 6803 (or simply *Synechocystis*) is the replacement of iron by copper in the Fe-S clusters of essential enzymes [40,41,42,43] and not the DNA damage mediated by copper-catalyzed production of ROS [42,44].

**Figure 1:**
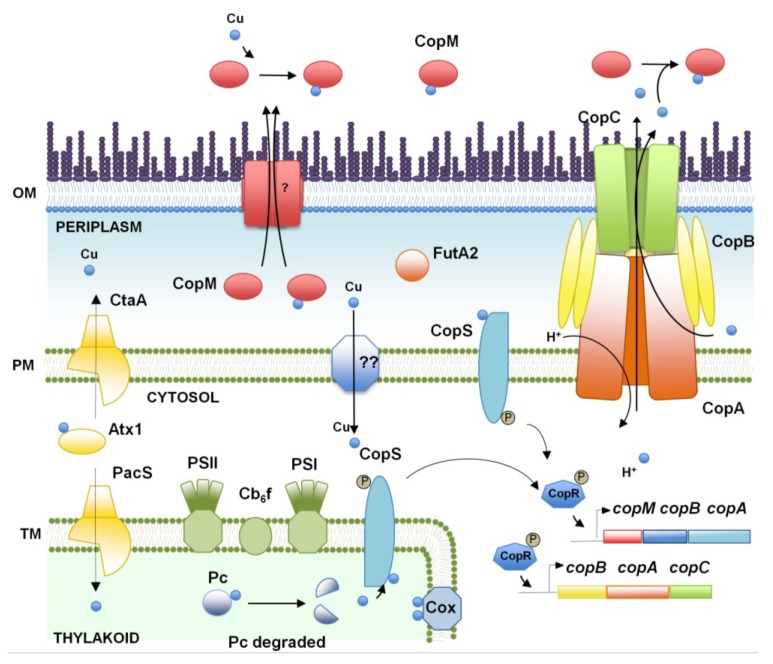
Schematic representation of the copper homeostasis mechanism in *Synechocystis* sp. PCC 6803. The proteins (and genes) mentioned in the figure are CopM (*sll0788* and *slr6039*), CopR (*sll0789* and *slr6040*), CopS (*sll0790* and *slr6041*), PacS (*sll1920*), CtaA (*slr1950*), Atx1 (*ssr2857*), FutA2 (*slr0513*), CopB (*slr6042*), CopA (*slr6043*), CopC (*slr6044*), Cox (cytochrome oxidase complex), Pc (*sll0199*), PSI (Photosystem I), PSII (Photosystem II) and Cb_6_f (cytochrome b_6_f). OM, outer membrane; PM, plasma membrane; TM, thylakoid membrane.

In bacteria, the first barrier of protection against copper is to localize all cuproproteins in the periplasm and the plasma membrane, avoiding the presence of this metal in the cytosol. Cyanobacteria are unusual among bacteria, as they require copper to enter into the cytosol to reach the thylakoid, their internal membrane system, which is discrete from the periplasm. Two essential proteins are present in the thylakoid: the blue-copper protein, plastocyanin, and the caa_3_-type cytochrome oxidase, which are involved in the photosynthetic and respiratory electron transport processes, respectively (Figure 1). This enzymatic demand for copper in the thylakoids sets cyanobacteria as an attractive model system to study copper trafficking to an internal compartment.

As for most of the metals, copper metabolism has been mainly analyzed in model cyanobacterium *Synechocystis*. Copper is imported in *Synechocystis* mediated by two P_I_-type ATPases, CtaA and PacS, which are located in the plasma and thylakoidal membranes, respectively. These two proteins are assisted by a small cytosolic soluble copper metallochaperone, Atx1 [42,45,46]. Copper import inside the cell is mediated by CtaA, which delivers it to Atx1 [42]; then, it is transferred to PacS, which finally transports it inside the thylakoid lumen (Figure 1). This model is supported by the data that shows that mutants in either *ctaA* or *pacS* render cells with reduced cytochrome caa_3_ oxidase and plastocyanin activities [45] and the lower internal copper quota of *ctaA* mutants. These phenotypes, along with the proposed location of PacS at the thylakoid membrane [47] and its requirement for copper tolerance, are in agreement with this proposed model. Although this pathway was widely accepted in the past, it is currently under discussion. Structural and biochemical analyses show that P_I_-type ATPases cannot import copper, and hence, CtaA and PacS can only efflux copper from the cell [48,49]. In fact, copper can reach plastocyanin in *pacS* and *atx1* mutant strains, but not in *ctaA* mutants, when cells are grown in standard copper-replete medium, suggesting that only CtaA is involved in the delivery to plastocyanin. This is in agreement with its low transport rate, as has been shown for other P_I_-ATPases involved in cuproprotein assembly [42,48,49]. Finally, both P_I_-type ATPases are required for CucA (a periplasmic copper-containing protein) to be loaded with copper, suggesting that they could also affect periplasmic copper metabolism [3]. This suggests that an additional copper import system should be present in *Synechocystis* (which will allow copper to enter the cytoplasm) and that both ATPases export copper to either the periplasm (CtaA) or both the periplasm and the thylakoid lumen (PacS). Because of this, the pathway for copper assembly in plastocyanin and caa_3_-type cytochrome oxidase could be similar to that of manganese loading in PSII, which seems to happen through the periplasm [50,51,52]. Another important protein related to copper import in *Synechocystis* is the periplasmic iron-binding protein, FutA2 [53]. Deletion of *futA2* leads to lower copper-dependent cytochrome caa_3_ oxidase activity and hyperaccumulation of copper in the periplasm. It is thought that FutA2 influences copper uptake into the cytosol by chelation of Fe(III), which, in the absence of FutA2, interferes with copper transport [53]. Transport through the outer membrane is also essential for metal homeostasis, and this is usually mediated by porins [54,55]. Although this has not been studied in *Synechocystis*, in *Anabaena* sp. PCC 7120 mutants, a TonB-dependent transporter (*iacT*, *all4026*), located at the outer membrane, affected the rate of copper transport. Furthermore, overexpression of this gene confers hypersensitivity to both copper and iron, suggesting that this protein is needed for copper and iron transport [56]. In marine environments, free copper is scarce, and most copper is complexed to an organic ligand that has been denoted, L_1_, a compound that has been shown to be produced by *Synechococcus* in response to copper [57]. In addition, *Anabaena* sp. PCC 7120 mutants affected in the production and transport of hydroxamate-based siderophore are also affected in copper transport [16,56,58].

Finally, one of the best known responses to copper availability in cyanobacteria is the replacement of cytochrome c_6_ by plastocyanin when copper is available [43,59,60,61,62]. Although this regulation has been known for a long time and the genes for both electron carriers are present in most cyanobacterial genomes (and most probably, its regulation is also conserved), nothing is known about the mechanisms of regulation. The appearance of plastocyanin probably conferred a selective advantage in iron-limited ecosystems, such as more oxidizing environments that were being generated by cyanobacteria through the released O_2_ by oxygenic photosynthesis [62].

Copper resistance has been mainly studied in *Synechocystis* and comprises a two-component system, CopRS, CopM, a periplasmic and extracellular copper binding protein [63], and an Heavy Metal Efflux-Resistance-Nodulation-Division (HME-RND) family export system, CopBAC (Figure 1, [29]). These genes are regulated by the presence of copper in the media through the CopRS two-component system. CopS is able to detect copper directly and probably activates CopR that is able to directly bind to the promoters of the *copMRS* and *copBAC* operons [29]. However, CopRS does not control the expression of any of the copper metabolism genes described above, such as *ctaA*, *pacS* and *atx1* [29,43]*.* Strains lacking any of these genes are more sensitive to copper, with *copRS* mutants being more sensitive than *copM* or *copBAC* mutants, suggesting that both CopM and CopBAC contribute to copper resistance [29,63]. These mutants also accumulate higher amounts of intracellular copper than the wild type. Moreover, CopS, the histidine kinase that detects copper and that belongs to the membrane-attached histidine kinases, is localized not only in the plasma membrane, but also in the thylakoid membrane. Therefore, CopS could be involved in copper detection in both the periplasm and the thylakoid lumen [29]. This could allow CopS not only to respond to conditions that alter extracellular copper supply, but also to those that alter the internal copper requirements, as almost (if not all) intracellular copper is localized to the thylakoids in plastocyanin and cytochrome cca_3_ oxidase. In fact, the *copMRS* operon is expressed in conditions that alter the photosynthetic electron transport and induced plastocyanin degradation (Figure 1, [29,64,65]). In *Anabaena* sp. PCC 7120 and *Synechococcus* sp. CC9311, operons coding for a putative RND transport system has been shown to be expressed in response to copper [34,56], although their contributions to copper resistance have not been analyzed. In contrast, two genes, coding for a membrane thioredoxin and an S-layer protein (and probably, the whole operon in which they are located), which were identified as copper induced in *Synechococcus* sp. CC9311, contribute to copper resistance and oxidative stress [34,66].

## 3. Nickel and Cobalt, Two Essential Metals for Cyanobacteria?

Nickel and cobalt are essential transition metals that are present at low levels, but play important roles in the cellular physiology of cyanobacteria [15,67]. Ni(II) is directly coordinated by proteins [68] or through the tetrapyrrole ring of coenzyme F_430_, which coordinates a nickel atom in methyl-coenzyme M reductase, while Co(II) is mainly used as a component in vitamin B_12_ [69]. In cyanobacteria, the major Ni-binding enzymes are urease and hydrogenases. Urease is needed to use urea as a nitrogen source [70] and probably also for arginine catabolism [71]. Urea appears to be a major nitrogen resource in the sea, and it has been suggested that both marine and freshwater cyanobacteria use it as a nitrogen source [72,73]; in some cases, the urease operon has been shown to be controlled by the nitrogen global regulator, NtcA [70,73,74]. The second Ni-containing enzymes are hydrogen-metabolizing/producing enzymes, named hydrogenases. Two distinct types are found in different cyanobacterial species: the bidirectional hydrogenases and the uptake hydrogenases. The bidirectional Ni-Fe hydrogenase (encoded by the *hox* genes, *hoxEFUYH*) can either take up or produce hydrogen and is found in both nitrogen fixing and non-fixing cyanobacterial genomes. This enzyme belongs to the NAD(P)-reducing class [75,76,77], although in *Synechocystis*, it has been recently described that it can be reduced by flavodoxin and ferredoxin [78]. This enzyme has great interest for the development of solar hydrogen production technologies using cyanobacteria. The second one is the uptake hydrogenase, which has the ability to recycle hydrogen (consuming O_2_) that is evolved as a side reaction during nitrogen fixation, since it is found in all nitrogen fixing species [79]. It is composed of small and large subunits encoded by the *hupS* and *hupL* genes, respectively. HupS mediates electron transport from the active site to the electron transport chain, and HupL contains two putative nickel-binding sites necessary for the coordination of the nickel in the active site [80,81]. Since this enzyme is an obstacle for hydrogen production, mutations disrupting the *hupSL* genes have been constructed in different cyanobacteria, and these mutant strains produce more hydrogen than the wild-type [82,83]. Other cyanobacterial Ni-enzymes described to date are Ni-superoxide dismutase [84] and glyoxalase I, a glutathione-dependent enzyme that requires Ni(II) and/or Co(II) for its activity [85,86]. Cobalt is mainly found in the corrin ring of coenzyme B_12_, a cofactor involved in both methyl group transfer and rearrangements reactions, and also in some non-corrin cobalt-containing enzymes, such as nitrile hydratase [69]. Although some cyanobacteria can synthesize vitamin B_12_-related compounds, others, like *Synechococcus* sp. PCC 7002, require B_12_ for growth. Little is known about how these compounds are used as cofactors in cobalamin-dependent enzymes beyond the essential enzyme, cobalamin-dependent methionine synthase [87].

Synthesis of Ni(II)/Co(II) enzymes and coenzyme B_12_ requires high-affinity uptake of the metal ions from natural environments, where they are available only in trace amounts most of the time. Nickel/cobalt transport is mediated by specific systems that belong to both secondary and primary transporters [15,88]. In general, the secondary systems found in cyanobacteria are related to nickel/cobalt transporters (NiCoTs) found in bacteria, which include the UreH transporters and HupE/UreJ permeases and which have been shown to mediate nickel transport. Most of the UreH proteins are predicted to function as high-affinity nickel transporters based on co-localization of these genes with those coding for NiFe hydrogenase, urease or nickel superoxide dismutase. UreH proteins are predicted to contain six transmembrane helices (TMH) and a histidine-rich segment in the cytoplasmic loop between TMH III and TMH IV. A member of the UreH family (called SodT) is found in some *Synechococcus* and all *Prochlorococcus* genomes and is predicted to contain six transmembrane domains [15,89]. *sodT* co-localizes with *sodN*, which code for a Ni-superoxide dismutase (SodN), and *sodX*, coding for a putative maturation peptidase, in all cyanobacterial genomes where they are present. SodT has been proposed to supply nickel for SodN, which is an essential protein in these organisms [15]. The HupE/UreJ proteins represent a secondary transporter that is widespread among bacteria, and they are encoded within certain NiFe hydrogenase and urease gene clusters. HupE/UreJ permeases are produced as a precursor and a mature protein that contain six transmembrane domains. Based on this genomic localization, a role in nickel uptake has been proposed. In the genome of marine cyanobacteria, a gene coding for a HupE transporter appears to be under the control of a B_12_-responsive riboswitch and probably involved in cobalt transport [90]. In the same way, a function in cobalt transport has been attributed to HupE in *Synechocystis*, because the *hupE* mutant growth defects were suppressed by adding cobalt or methionine [91]. Primary active nickel importers contain subunits with an ATP binding cassette (ABC) and are driven by ATP hydrolysis. Bioinformatics studies using 19 cyanobacterial genomes only found the CbiMNQO/NikMNQO systems as this primary cobalt/nickel transporters [92,93]. These genes code for a member of the energy-coupling factor (ECF) family of micronutrient importers and consist of a conserved transmembrane protein (Nik/CbiQ), an ABC ATPases (Nik/CbiO) [93] and a component that is responsible for substrate specificity (Nik/CbiMN) [92]. CbiMNQO systems have been annotated as cobalt uptake systems based on their genomic co-localization with B_12_ biosynthesis genes or by the presence of the regulatory B_12_ riboswitch.

The systems described above imply routes that are used by bacteria to import nickel and cobalt across the inner membrane to the cytosol, but metals need to also enter the periplasm through the outer membrane. As has been discussed for copper, this transport is usually carried out by porins [54,55] and a TonB-dependent transporter. In Proteobacteria, nickel-regulated genes for outer-membrane transporters, ExbB-EsbD-TonB clusters, have been identified [93]. Putative TonB-dependent transporters, implicated in the transport of cobalamin and metals, such as nickel, are predicted in bioinformatics studies based on the sequence similarity of these systems in cyanobacteria [87]. In *Anabaena* sp. PCC 7120, several TonB-dependent receptors genes have been shown to change its expression levels in response to metal and nitrogen source availability, which suggest that they could be involved in transporting different metals across the outer membrane [56,58,94,95].

These import system must be tightly regulated in response to metal availability and cellular requirements/demands. The known nickel regulatory systems used in bacteria to regulate nickel import, such as NikR and Nur [96,97], are absent from cyanobacterial genomes, implying that a novel form of nickel transport regulation should exist. The cobalt regulatory system is also unknown, but as has been indicated above, several transporters are associated with a putative B_12_ riboswitch, suggesting a role in cobalt uptake and coordination with the synthesis of coenzyme B_12_ [89].

Finally, an excess of nickel/cobalt that could cause a perturbation of protein function is counterbalanced by the presence of efflux pumps, which expel these metal ions out of the cell. In addition, there could be other proteins involved in metal tolerance. In this regard, a global proteomic approach has revealed that the chaperones, HspA and GroES, are specifically upregulated in the presence of high concentrations of nickel and cobalt, respectively [8]. These data suggest that these chaperones may be directly involved in the tolerance for these metals. Furthermore, it also has been shown that FutA2, a periplasmic iron-binding protein that seems to be essential for metal homeostasis [53], is downregulated in the presence of both nickel and cobalt [8], pointing to cross-talk between different metal metabolisms.

**Figure 2:**
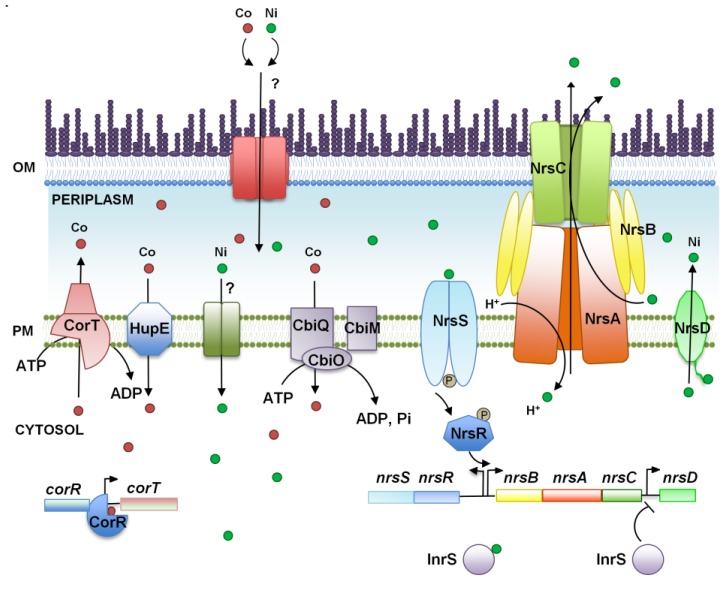
Schematic representation of the cobalt and nickel homeostasis mechanisms in *Synechocystis* sp. PCC 6803. The proteins (and genes) mentioned in the figure are CorT or CoaT (*slr0797*), CorR or CoaR (*sll0794*), NrsB (*slr0793*), NrsA (*slr0794*), NrsC (*slr0795*), NrsD (*slr0796*), InrS (*sll0176*), HupE (*slr2135*), NrsR (*sll0797*), NrsS (*sll0798*), CbiO (*sll0384*), CbiQ (*sll0385*) and CbiM (*sll0383*). OM, outer membrane; PM, plasma membrane.

As for the other metals, nickel and cobalt resistance systems have been mainly studied in *Synechocystis* (Figure 2). The genes involved in the resistance to these two metals are part of a metal-regulated gene cluster involved in resistance against zinc, copper, cobalt and nickel [28,29,31,32]. This 14-kb region consists of 14 ORFs organized into seven putative transcriptional units: the *nrsBACD* operon involved in nickel tolerance and regulated by the upstream *nrsRS* operon [28,98], *corT* (*coaA*), encoding a putative co-translocating P_I_-type ATPase under the regulation of the upstream *corR* (*coaR*) product [28,31], *ziaA*, encoding a putative Zn-efflux P_I_-type ATPase and regulated by the product of *ziaR* [32], and the *copMRS* operon, which is involved in copper resistance (see above, [29]).

The cobalt resistance system is composed of CorR (or CoaR), a MerR homolog that has been identified as activator of the *corT* (*coaT*) gene, which codes for a co-dependent P_I_-type ATPase [28,31]. This transcription factor contains a precorrin isomerase domain and responds to vitamin B_12_ intermediaries [31,99], suggesting that, in addition, to *coaT* (*corT*) activation in response to cobalt excess, it could be also involved in coordinating cobalt transport and vitamin B_12_ synthesis. Mutation of any of these genes confers sensitivity to cobalt excess in the media, although its effects on vitamin B_12_ metabolism were not analyzed [28,31].

The *nrsBACD* operon encodes two putative nickel efflux systems: an RND efflux system (encoded *nrsBAC* genes) and a major facilitator superfamily (MFS) permease (encoded by *nrsD*) that contains a metal binding domain (histidine-rich region) in its C-terminus [28]. A two-component signal transduction system (encoded by the *nrsRS* genes, also known as Hik30-Rre33) controls the Ni-dependent induction of the *nrsBACD* operon and its own expression [98]. NrsS is a histidine kinase sensor with an N-terminal periplasmic domain that shares sequence similarity with the alpha subunit of the methyl-coenzyme M reductase (MCR) from Methanobacteria (an enzyme able to bind nickel through a F_430_ cofactor, suggesting that this domain could bind Ni(II) using a related structure). This histidine kinase senses nickel and modulates the Ni(II) efflux operon via the DNA-binding transcriptional regulator, NrsR, a response regulator of the PhoB/OmpR family [98]. Mutants in any of these genes are also more sensitive than the wild-type to nickel excess [28,98]. *nrsD*, the last gene of the *nrsBACD* operon, has its own promoter between the final two ORFs in the *nrsBACD* operon, and it has been recently shown to be also regulated by InrS (for internal nickel-responsive sensor), a CsoR/RcnR-like transcriptional repressor [100]. This family of transcription factors were identified as copper responsive [97,101] and InrS contains conserved copper binding residues. *In vitro*, InrS is able to bind Cu(I) more tightly than Ni(II), although it was proposed that this protein does not have access to copper *in vivo* under steady-state conditions, because copper is completely buffered in *Synechocystis*’ cytoplasm [42]. Later, it was shown that the *nrsD* gene is also induced by copper (and Zn) after a short challenge [102], although neither *nrsBAC* nor *nrsRS* genes were induced by copper [43]. InrS^−^ mutants grow poorly in the absence of added nickel [43,100], probably because they contain lower internal nickel quota [100], suggesting that this metal is essential for *Synechocystis* growth. This phenotype is suppressed in a double-mutant lacking also *nrsD* (InrS^−^ NrsD^−^), but surprisingly was shown extremely sensitive to different metals, suggesting that InrS could have a central role in metal homeostasis, probably controlling other elements [43].

## 4. Arsenic, a World Wide Spread Phosphate Competitor

Arsenic is an ubiquitous toxic metalloid that causes serious health problems in many places of the world where the arsenic content of drinking water is well over the recommended limits [103]. Arsenic is present in two biologically active forms, arsenate [As^V^] and arsenite [As^III^], depending on the redox potential of the environment. Arsenate is a phosphate analogue and, therefore, enters the cells through phosphate transporters. Its toxicity is mediated by replacing phosphate in essential biochemical reactions, such as oxidative phosphorylation and glycolysis. The resulting arseno-compounds are extremely labile and hydrolyze spontaneously at millisecond rates, making them unable to be used by living organisms [104,105,106,107]. On the other hand, arsenite enters the cell through aquaglyceroporins [108,109,110] and exerts its toxicity through binding to dithiols, forming arsenothiols that disrupt protein function and that ultimately generate reactive oxygen species (ROS) [108,109,110,111]. Because of the high affinity for sulfur, arsenite is able to bind to the main redox buffer in the cells, glutathione (GSH), forming As^III^-GSH_2_, and depletes its pool, thus contributing to ROS generation. Despite being toxic, arsenic is also used by some microorganisms as an electron acceptor in the anaerobic respiratory chain, as an electron donor to grow chemo-lithotrophically and even for anoxygenic photosynthesis by some photosynthetic bacteria and cyanobacteria [112,113]. It has been postulated that arsenic compounds played an important role during early life on Earth before the appearance of molecular oxygen as an electron acceptor and donor [113,114].

Because of the extensive use and distribution of arsenic compounds, arsenic resistance is widespread among living organisms. Many resistance systems consist of the reduction of arsenate to arsenite, followed by export of the latter outside the cell or its transport to the vacuole. Another detoxification system that is present from bacteria to animals is arsenic methylation, which conjugates arsenic to methyl groups and can lead to the formation of arsenic volatile species [115,116].

**Figure 3:**
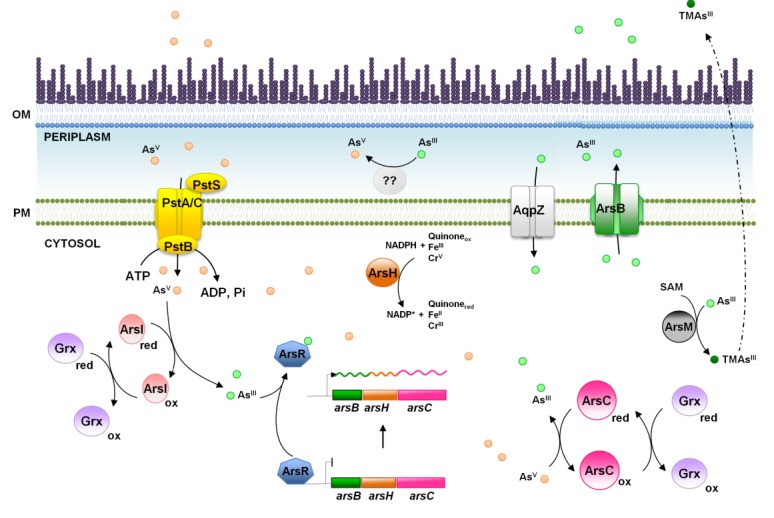
Schematic representation of the arsenic resistance mechanism in *Synechocystis* sp. PCC 6803. The proteins (and genes) mentioned in the figure are ArsR (*sll1957*), ArsB (*slr0944*), ArsH (*slr0945*), ArsC (*slr0946*), ArsI (*sll5104* and *slr6037*), Grx (GrxA or GrxB; *ssr2061* and *slr1562*), ArsM (*slr0303*), AqpZ (aquaporin; *slr2057*), PstA (*sll0682* and *slr1242*), PstB (*sll0683*-*84* and *slr1250*), PstC (*sll0681* and *slr1248*) and PstS (*sll0680*, *sll0679*, *slr1247* and *slr0540*). OM, outer membrane; PM, plasma membrane.

In cyanobacteria, arsenic metabolism and resistance is best understood in *Synechocystis* (Figure 3). The main arsenic resistance mechanism is mediated by an operon of three genes (*arsBHC*) that is regulated by an unlinked *arsR* [30,117]. The operon includes an Acr3-arsenite transporter gene, *arsB*, *arsH*, which codes for a NADPH-FMN reductase, able to reduce several compounds without a clear function in arsenic resistance [30,118,119], and an arsenate reductase gene, *arsC*. At least two other resistance determinants have been described in *Synechocystis*: an additional arsenate reductase from the *E. coli* family (encoded by two nearly identical genes, *arsI1* and *arsI2* [120]) and an arsenite methylase gene, *arsM* [121]. ArsI is only essential for arsenic resistance in the absence of ArsC, probably due to its low level of expression [117,120]. Furthermore, global studies have shown that arsenic induces a strong oxidative stress response, disrupts metal homeostasis and depletes the GSH pool [117,122,123], which is in agreement with what has been observed in other organisms [111].

Arsenate reduction to arsenite is catalyzed by arsenate reductase, an enzymatic activity carried out by at least three non-related families of enzymes that use thioredoxin, glutaredoxin or mycoredoxin as electron donors [120,124,125,126]. Two types of arsenate reductases appear in cyanobacteria encoded by *arsC* and *arsI* genes in *Synechocystis*. *arsC* codes for a new type of hybrid arsenate reductase exclusively found in cyanobacteria, that although related to thioredoxin-dependent ones, uses the glutathione/glutaredoxin system for reduction [30,120,124], while *arsI* genes code for *E. coli*-like glutaredoxin-dependent arsenate reductases [30,120]. Although the ArsC_Syn_ is exclusively found in cyanobacteria, many genomes also contain genes coding for *E. coli*-like arsenate reductases [120,127]. The fact that all arsenate reductases present in cyanobacteria are glutaredoxin dependent could be related to the essentiality of the thioredoxin system in these organisms [128,129], which has forced them to find alternative reductants for these enzymes.

Arsenite export is mediated by two families of proteins: ArsB proteins, which are present only in bacteria [130], and Acr3 proteins, found in different organisms, including bacteria, fungi and plants [131,132]. Many cyanobacterial genomes contain arsenic resistance gene clusters with the ACR3 family of transporters and arsenate reductase genes (of either ArsC_Syn_ or the *E. coli*-type) [120,127]. In contrast, no homologs of the *E. coli* ArsB protein can be easily identified in them.

ArsM from several cyanobacteria is able to methylate arsenite to the volatile trimethylarsine [TMA(III)] using S-adenosyl methionine and glutathione as methyl donors *in vitro* [121], and the *arsM* gene is present in many cyanobacterial genomes. Although the role of ArsM in arsenic resistance has not been tested *in vivo* in cyanobacteria, *E. coli* strains carrying *arsM* genes from different cyanobacteria are more resistant to arsenite [121]. Furthermore, methylated arsenic forms are abundant in the oceans [133], and methylated arsenic species are detected when several cyanobacterial strains are treated with arsenic [121,134,135], suggesting that arsenic methylation could play a role in resistance. On the other hand, *arsM* expression has been reported to be both upregulated by arsenic after two weeks of exposure [121] or not regulated after one hour of both arsenate and arsenite treatments [117]. Although the conditions were completely different, what seems to be clear is that the *arsM* gene is not regulated by the transcriptional repressor, ArsR, at least in *Synechocystis* [117].

**Figure 4:**
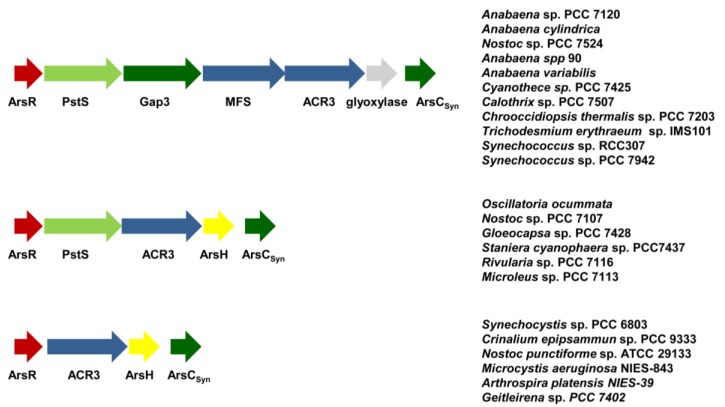
Genomic organization of arsenic resistance genes in several cyanobacteria.

Bioinformatics analyses have shown that arsenic resistance genes are widespread in most sequenced cyanobacterial genomes [120,127]. Inspection of the genomic context have allowed us to identify four additional genes associated with arsenic resistance genes: a periplasmic phosphate binding protein (PstS), a glyceraldehyde-3-phosphate dehydrogenase (Gap3), a major facilitator superfamily transporter (MFS) and a putative glyoxalase, which are probably organized forming an operon with *arsB* (ACR3) and *arsC* (ArsC_Syn_) genes (Figure 4). In some cases, an ArsR coding gene is also localized upstream of the PstS coding gene, strongly suggesting that these genes are co-regulated in response to arsenic. The function of these proteins in arsenic resistance remains to be determined, but it is possible that they could influence phosphate transport (PstS and MFS transporter), be arsenate insensitive isoforms (PstS or Gap3) or constitute a new arsenic resistance mechanism transporting arsenic outside the cell (MFS or PtsS or the glyoxalase).

Phosphate has a great influence on arsenic resistance in cyanobacteria, because the phosphate concentration interferes with arsenate transport. The phosphate transport system (which is the main arsenate transport system) is induced under phosphate deprivation [136,137], therefore increasing arsenate transport [134,135,138,139,140,141]. Furthermore, phosphate starvation also induced *arsB* expression in *Crocosphaera* *watsonii* [137], and arsenic represses phosphate transport gene expression in *Synechocystis* [117]. In addition, the specificity and selectivity of the phosphate transport system greatly influence arsenate transport [142]. Phosphate also influences the redox state of the arsenic that accumulated in the media after it is metabolized by cyanobacteria. In *Synechocystis* sp. PCC 6803, arsenate is the main form accumulated in the media after long-term exposure to both arsenite and arsenate in phosphate-rich conditions, but arsenite accumulates in phosphate-depleted media [134]. This is a consequence of the active arsenate reduction in the cytoplasm, which efficiently reduces all arsenate to arsenite, which is extruded outside the cells. Once outside, this arsenite is oxidized to arsenate, which, under phosphate-replete conditions, is not transported inside the cells, while under phosphate-deficient conditions, any arsenate that is produced is transported into the cells and reduced. The mechanism for extracellular arsenite oxidation is not clear and remains to be elucidated [134].

Finally, phytochelatins, which are GSH polymers (*n* = 2–16), are well known to contribute to arsenite resistance [143]. Although genes with homology to phytochelatin synthase are present in some cyanobacteria [144] and phytochelatin contents increase in response to arsenic [122], it is not known whether phytochelatin is able to confer arsenic resistance.

## 5. Conclusions

This review highlights our understanding of metal homeostasis systems in cyanobacteria and how metal transporters, metal sensing and regulators are selective for one (or two) metal ion(s). This cellular-level approach is necessary to establish accurately the mechanism involved in the interaction between cyanobacteria and metals under deficiency or toxic concentration conditions. As discussed above, this information may be used for the design of promising detector systems for metal bioremediation or for the determination of the optimal growth conditions in order to use them as biofactories. Nevertheless, it is becoming clear that metal metabolism, as occurs with many cellular process, is interconnected. In addition, natural ecosystems are usually limited by several nutritional factors, including the availability of essential metals, like iron (this limitation is a key point for ocean productivity). The excess of metals happens frequently in nature, in many cases due to contamination produced by human activity. This type of contamination includes more than one metal, as they are in general more soluble in acid environments, for example mine drainage or the chemical industry. Furthermore, the nutrition status for one metal can greatly determine the responses to other metal excesses or deficiencies, as has been shown for Zn starved *Chlamydomonas* *reinhardtii*, which show symptoms of copper deficiency and accumulate higher intracellular amounts of Mn and Fe [145]. This highlights the importance of studying the metal responses to different combinations of metal nutrition statuses in the lab to be able to understand what happens in natural systems.
